# The Health Professional’s View on the Inclusion of Age in the Recommendations for Pneumococcal Vaccination: Results of a Cross-Sectional Survey in France

**DOI:** 10.3390/geriatrics7010004

**Published:** 2021-12-23

**Authors:** Gaëlle Farge, Benoît de Wazières, Jocelyn Raude, Clémence Delavelle, Fabienne Humbert, Cécile Janssen

**Affiliations:** 1 MSD Vaccins, 69007 Lyon, France; gaelle.farge@msd.com (G.F.); clemence.delavelle@msd.com (C.D.); fabienne.humbert@msd.com (F.H.); 2 Service de Médecine Interne et Gériatrique, CHU de Nîmes, 30900 Nîmes, France; 3 École des Hautes Études en Santé Publique, 35043 Rennes, France; Jocelyn.Raude@ehesp.fr; 4 Service de Maladies Infectieuses Médecine Interne, CH Annecy-Genevois, 74374 Epagny Metz-Tessy, France; cjanssen@ch-annecygenevois.fr

**Keywords:** *Streptococcus pneumoniae*, invasive pneumococcal disease, vaccination, age, public health

## Abstract

Elderly people are at high risk for pneumococcal infections. However, older age is not an eligibility factor for pneumococcal vaccination in France. Adults with certain co-morbidities or immunocompromised states are eligible for vaccination, which leaves adults aged ≥65 years without comorbidities at-risk for pneumococcal infections. The objective of the study was to evaluate the acceptability to healthcare professionals (HCPs) of extending pneumococcal vaccination to all individuals ≥65 years. Based on themes identified in semi-structured interviews with 24 HCPs, a representative sample of 500 general practitioners and pharmacists were surveyed about their knowledge, attitudes and beliefs with respect to pneumococcal vaccination for individuals ≥65 years. Current recommendations for pneumococcal vaccination are poorly understood by participants (mean score: 5.8/10). Respondents were generally supportive of inclusion of age in vaccination recommendations (7.5/10), with 58% being very supportive. For 72% of HCPs, this would contribute to improved vaccination coverage. The strategy could be facilitated by associating pneumococcal vaccination with the influenza vaccination campaign (8.3/10). Pharmacists were favourable to participating in pneumococcal vaccination (8.5/10). In conclusion, extension of pneumococcal vaccination to all people aged ≥65 years would be welcomed by HCPs, simplifying identification of patients to be vaccinated and potentially improving vaccination coverage.

## 1. Introduction

*Streptococcus pneumoniae* is a commensal bacterium of the human nasopharynx which is the principal cause of community acquired pneumonia (CAP) [1,2]. Non-invasive pneumococcal infections may also present as sinusitis or otitis media [1]. In around 25% of cases, invasive pneumococcal disease (IPD) may develop, leading to meningitis or bacteraemia. In Europe, the incidence of IPD in the general population has been estimated to be between 10 and 30 cases per 100,000 and that of CAP between 100 and 1000 cases per 100,000, although incidence rates are highly age-dependent [1,2]. Nonetheless CAP and IPD carry high morbidity and mortality [2], with a case fatality rate in high-income countries of between ten and thirty percent [1]. Notably, pneumococcal disease is becoming of increasing concern in older individuals [1]. Indeed, recent data from the Epibac observatory indicate that 55% of cases of IPD in France occur in the ≥65-year age group [3]. A study conducted in the French national insurance claims database reported that >8000 people aged ≥50 years were hospitalised for community-acquired pneumococcal pneumonia in 2014, with an in-hospital mortality rate of around 10% and a one-year mortality rate of around 20% [4].

Despite the introduction of pneumococcal conjugate and polysaccharide vaccines to the national immunisation programs (NIPs) of many countries, inadequate vaccination coverage means that pneumococcal disease (PD) is still associated with substantial mortality and morbidity, especially in older adults. One of the challenges of pneumococcal vaccination is the multiplicity of different strains of the pathogen, with over ninety serotypes of *S. pneumoniae* identified to date [5]. Currently, 13-valent (PCV13; Prevenar 13^®^) and 23-valent (PPSV23; Pneumovax^®^) are the only vaccines currently approved in France for the prevention of PD in adults.

Most European countries have implemented NIPs for pneumococcal vaccination, although the risk groups recommended for vaccination vary between countries [6]. Recommendations and reimbursement eligibility generally target, in addition to young children, older adults (aged ≥60 or ≥65 years) and adults with certain severe chronic diseases (such as diabetes, COPD or heart disease) and with compromised immune function, such as those with HIV infections or asplenia, or those receiving immunosuppressant therapy [6]. Although the majority of OECD (Organisation for Economic Co-operation and Development) countries include age as an eligibility factor for vaccination against *S. pneumoniae* [6], France is one of the rare European countries not to recommend systematic vaccination of all older adults, regardless of their health status [6]. In 2017, the National Immunisation Technical Advisory Group of the French public health authority decided to recommend pneumococcal vaccination only for individuals with certain severe chronic diseases (such as diabetes, COPD or heart disease), and with compromised immune function, not considering individuals aged from 65 to 84 years, principally on the grounds that it might not be cost-effective [7].

Vaccination coverage in adults in France is extremely low (between 5% and 20%) and varies considerably between the different risk groups [8]. As the risk of pneumococcal diseases is very high in adults aged ≥65 years, this represents a significant loss of opportunity. A number of factors may contribute to this unsatisfactory situation [8]. Firstly, identifying patients eligible for vaccination is not straightforward, since different comorbidities may be managed by different specialists or general practitioners. Secondly, who should be responsible for assessing whether the patient fulfils the criteria is not clearly defined. In order to improve pneumococcal vaccination coverage, a number of different strategies have been proposed [8]. These include simplification of the vaccination schedule, extension of vaccination to all individuals aged ≥65 years regardless of comorbidities, or involvement of other HCPs such as pharmacists in the vaccination strategy. However, the impact of any change in the recommended vaccination strategy will depend on the adherence of HCPs to implementation of the new strategy. For this reason, it is important to evaluate their perceptions of any potential changes to the recommendations beforehand. The objective of the present study was to evaluate attitudes of healthcare professionals (HCPs) to extending pneumococcal vaccination to all elderly patients and to how this could be achieved.

## 2. Methods

This cross-sectional study was carried out through complementary qualitative and quantitative approaches. The qualitative phase, conducted between September and December 2020, consisted of interviews with physicians involved in prevention or management of PD. The information from the qualitative interviews was used to identify themes to be explored in the survey questionnaire. The quantitative phase consisted of a web-based survey of 500 GPs and pharmacists in France using a self-administered questionnaire, conducted between January and March 2021. The study was performed by a Survey Institute (IPSOS Healthcare, Paris, France) using an established panel of HCPs. The data from the quantitative phase of the survey is presented in this report.

### 2.1. Study Participants

Participants were recruited through an existing web-based healthcare panel (SERMO Panel; IPSOS Healthcare, Paris, France). Potential participants were identified by a random sampling procedure stratified by specialty, age, gender and region. A quota method was applied to ensure demographic representativeness of the sample with respect to all French GPs or pharmacists using data from the national economic statistics agency (DREES). The target sample consisted of 300 GPs and 200 retail pharmacists. Selected HCPs were asked to complete a screening questionnaire to ensure that the eligibility criteria were fulfilled. These criteria included age between 30 and 65 years, at least two years of medical experience, with at least 25% of their patients being aged ≥65 years, and active involvement in vaccination delivery or recommendation. These criteria were essentially pragmatic ones to ensure that the HCPs enrolled had current experience of vaccinating elderly people. The screening questionnaire also included a conflict-of-interest disclosure.

### 2.2. Qualitative Phase

Semi-structured qualitative anonymous interviews were conducted with HCPs from different medical specialties in order to identify the main themes and evaluation criteria for building the quantitative questionnaire. The sample was intentionally diverse in order to be as exhaustive as possible in terms of topics and attitudes described by participants. It included GPs, retail pharmacists, chest physicians, geriatricians and infectious disease specialists. Interviews were prearranged and carried out by telephone by a trained interviewer from IPSOS and recorded. Interviews lasted approximately 45–60 min each. The interview started with a series of open questions inviting participants to talk freely about their perceptions of IPD, its severity and its importance from a public health perspective, and what they discussed concerning this with their patients. The next part of the interview was devoted to more focused questions concerning their attitudes to vaccination. Participants were next asked what they thought of the current national vaccination recommendations, about whether they thought these needed to be updated and, if so, in which way. Finally, participants were invited to comment on possible ways to improve vaccination coverage, based on practice in other countries, or strategies for other vaccines in France. Information from the interviews was aggregated and a thematic analysis performed to identify material for the final questionnaire.

### 2.3. Quantitative Phase: Study Questionnaire

The final questionnaire included 35 questions, some of which had subsidiary questions, and took around thirty minutes to complete. The questionnaire was addressed to participants and completed using a dedicated web-based interface. Respondents could not skip questions. Different types of questions were asked, including closed binary choice questions (response options: yes or no), closed multiple response questions and Likert-type questions using 10-point ordinal rating scales. Data on sociodemographic characteristics, geographical location and clinical practice environment were collected for all participants.

Questions were divided into several broad themes, including the level of awareness of HCPs with respect to pneumococcal disease, their perceptions of current pneumococcal vaccination strategy and current vaccination practice. The acceptability of revising the pneumococcal vaccination recommendations to include age as a criterion for vaccination was evaluated. Participants were also asked to evaluate a number of statements pertaining to each of these scenarios on a five-point Likert scale (don’t know, don’t agree at all, agree a little, partially agree and fully agree). In addition, participants were asked their opinion on three potential ways to support such an extension of the age criterion for vaccination. Three such measures, which had been identified during the qualitative phase of the study, were proposed. The first was to use the medical visit that all people in France are encouraged to attend [9] when they retire in order to communicate the importance of pneumococcal vaccination. The second was to use the annual national influenza vaccination campaign for people aged ≥65 years to provide a voucher for pneumococcal vaccination. The third measure was to extend pneumococcal vaccination competence to pharmacists. Participants were asked to rate each of these with respect to relevance, feasibility, credibility and likelihood that they would increase vaccination coverage in elderly people. The mean score on these items were used to calculate a composite score reflecting whether each measure would be likely to facilitate the extension of the vaccination criteria to include age.

### 2.4. Statistical Analysis

For the binary choice and multiple response questions, data are presented as the number and percentage of participants choosing each response option. For the ordinal rating scales, results are presented either as a mean score with its standard deviation (SD) or as a percentage of participants providing scores in the range of 1–3, 4–7 and 8–10. All data were analysed by HCP speciality (GPs only and pharmacists only) and pooled for both types of HCP. Score distributions were compared between GPs and pharmacists using Student’s *t*-test. In the presentation of the results, scores are provided separately for GPs and pharmacists only when there was a statistically significant difference (*p* < 0.05) between the two groups. Associations between disease awareness and HCP attitudes were evaluated using the χ^2^ test. Data were analysed using Quantum software (San José, CA, USA).

### 2.5. Ethics

The survey was conducted in accordance with the ESOMAR International Code on Market and Social Practice, the EphMRA Code of Conduct, relevant national and international European legislation on medical research, and Good Pharmacoepidemiologic Practice guidelines. Use of the IPSOS HCP panel for medical research has been approved by the French national committee in charge of personal data protection (Commission Nationale de l’Informatique et des Libertés; CNIL). In the screening questionnaire, all participants had to give their consent to the collection and analysis of their data.

## 3. Results

### 3.1. Qualitative Phase

The qualitative phase consisted of interviews with 24 HCPs (4 pharmacists, 5 GPs, 4 geriatricians, 5 chest physicians, 5 infectious disease specialists, 1 PD expert). Disease-related themes identified included understanding of risk factors by HCPs, the importance of vaccination, understanding of the prevalence and severity of PD by HCPs, and public awareness of PD. Vaccine-related themes included the type of vaccines available, the recommended vaccination schedule, the need for simplified recommendations and the appropriateness of the target population. Ways forward identified included involving pharmacists in the vaccination programme and linking pneumococcal vaccination with influenza vaccination.

### 3.2. Study Participants

Overall, 301 GPs and 200 pharmacists participated in the study. Their demographic characteristics are presented in Table 1. For the GPs, around one-third of their patients were aged >65 years.

### 3.3. Level of Disease Awareness

When asked to rate their level of knowledge of *S*. *pneumoniae* infections on a scale of 1 (very limited) to 10 (very well-informed), the mean score was 6.6 ± 1.4 for GPs and 4.7 ± 1.7 for pharmacists. Only 24 HCPs (4.8%) correctly identified all diseases potentially attributable to *S. pneumoniae* from a proposed list of fifteen diseases. However, most HCPs recognised the potential severity of PD (7.9 ± 1.2) and 90.8% (*N* = 455) considered that the severity of these infections varied with age.

### 3.4. Perception of Current Pneumococcal Vaccination Strategy

Asked to rate their level of knowledge of pneumococcal vaccination on a scale of 1 (very limited) to 10 (very well-informed), the mean score was 7.2 ± 1.5 for GPs and 5.9 ± 1.8 for pharmacists. Current French national recommendations for pneumococcal vaccination are not well known by HCPs (6.6 ± 1.6 for GPs and 4.6 ± 2.0 for pharmacists). Currently, 63.8% of GPs (*N* = 192) take into account the age of the patient when recommending pneumococcal vaccination, to the same extent as the presence of chronic diseases (78.4%; *N* = 236) and compromised immune function (73.1%; *N* = 220). In addition, 24.9% of GPs (*N* = 75) and 34.0% of pharmacists (*N* = 68) incorrectly consider that the French health authorities (HAS) already recommend that all individuals aged ≥65 years be vaccinated against PI. Overall, the recommendations were considered to be effective and easy to apply, although there was less agreement on whether they were clear and easy to remember (data not shown).

General practitioners were generally aware of the two commercially available pneumococcal vaccines in France (8.2 ± 1.5 for PCV13 and 8.0 ± 1.5 for PPSV23), although this was less the case for pharmacists (6.9 ± 1.8 and 6.6 ± 1.9 respectively). Only 130 GPs (43.2%) declared that they recommend pneumococcal vaccination very often and 88 (29.2%) declared that they strictly adhered to the recommendations of the French health authorities for vaccination.

### 3.5. Acceptability of Including Age as a Criterion for Vaccination

**Figure 1:**
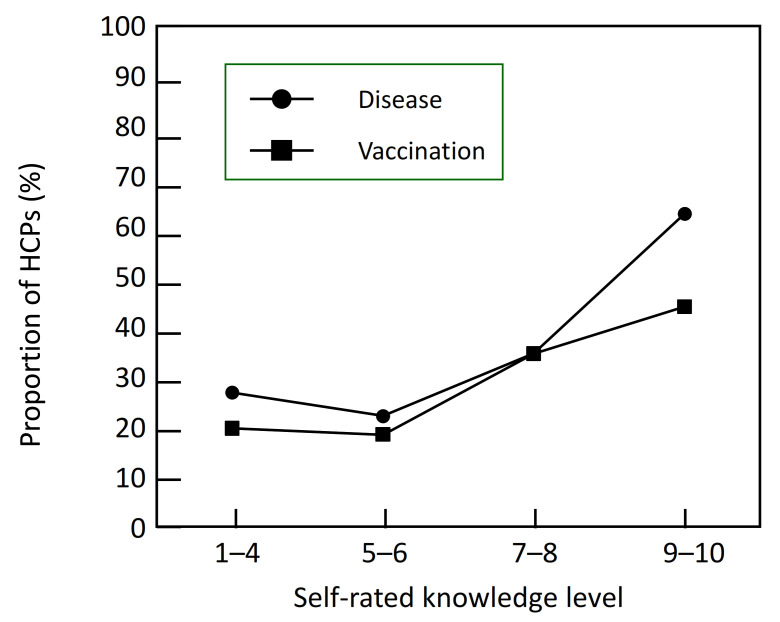
Acceptability of including age in vaccination recommendations as a function of disease awareness. Data are presented as the proportion of participants who fully agreed with the extension of vaccination criteria to include age (rating score of 9 or 10), as a function of how they rated their knowledge of pneumococcal disease or pneumococcal vaccination. Data are combined for GPs and pharmacists.

Pharmacists and GPs were generally likely to support the inclusion of age in HAS recommendations (7.5 ± 1.8), with 291 (58.1%) being very supportive (≥8/10). The level of agreement with extending the criteria for vaccination was correlated with how knowledgeable the HCPs considered themselves about pneumococcal disease (*p* = 0.0004; χ^2^ test) and its prevention (*p* = 0.0026; χ^2^ test), with the most knowledgeable being more frequently in agreement (Figure 1). When asked what would be the most appropriate age at which to recommend vaccination, 63.7% of HCPs (*N* = 319) recommended starting from the age of 60, with a median age of 60.4 years.

When asked to select from a pre-specified list of potential benefits that could be gained from including age as a specific criterion for pneumococcal recommendation, the most frequently cited item was an improvement of the vaccination coverage (provided in the Supplementary Table S1). The principal barriers to implementation of the strategy were perceived to be vaccine hesitancy in a segment of the French population and lack of consent from elderly patients (provided in the Supplementary Table S1).

**Figure 2:**
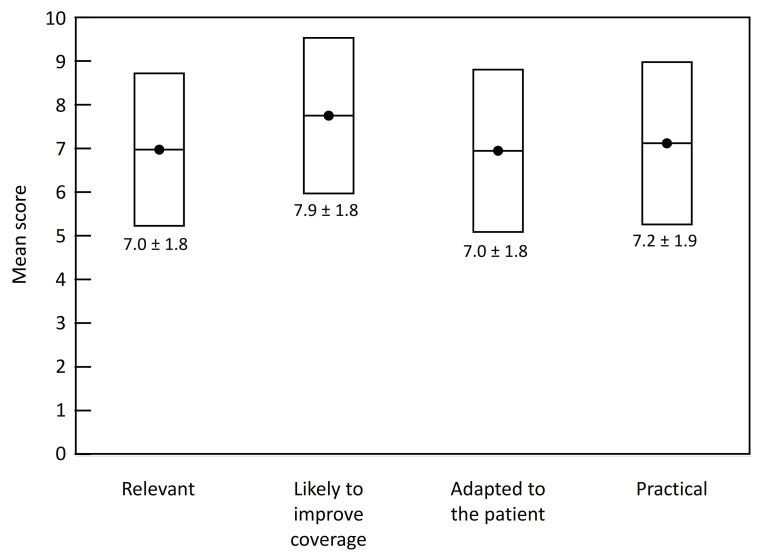
Acceptability of vaccination with PPSV23 alone in healthy individuals ≥65 years. Data are presented as box plots representing mean scores on a scale from 0 (completely disagree) to 10 (fully agree), with their standard deviations. Data from GPs and pharmacists have been pooled.

With respect to vaccination strategy, 71% of HCPs considered that vaccination with PPSV23 alone would be suitable in healthy individuals aged ≥65 years, with the sequential scheme (PCV13 followed by PPSV23) maintained in those with chronic diseases or who are immunocompromised. Mean scores for the relevance of this strategy, its potential to increase vaccination coverage, its suitably for patients and its feasibility ranged between 6.9 and 8.1 (Figure 2).

### 3.6. Supporting Measures

Rating scores for the different potential accompanying measures to support the extension of the vaccination criteria to include age are presented in Table 2.

The strategy of pairing pneumococcal vaccination with annual influenza vaccination campaigns (through distribution of a care voucher) was ranked favourably. An overall acceptability score was calculated as the mean of the individual scores for relevance, credibility, ease of implementation and impact on vaccination coverage. This overall score was 8.4 ± 1.7 for GPs and pharmacists combined. In both groups, over 90% of participants agreed that it would increase vaccination coverage, that it could be integrated into an existing public health initiative and that it would profit from awareness of the influenza campaign by HCPs and the general public.

Regarding involving pharmacists in pneumococcal vaccination, the opinions of GPs and pharmacists diverged with a mean acceptability score of 5.7 ± 2.6 for GPs and of 8.5 ± 7.0 for pharmacists. Only 43.5% of GPs (*N* = 131) fully or partially agreed with the statement that pharmacists were competent for administering vaccines, compared to 91.5% of pharmacists (*N* = 183). Similarly, only 52.5% of GPs (*N* = 158) but 97.5% of pharmacists (*N* = 195) agreed that the pharmacist had generally built up a relationship of trust with the patient. In addition, only 33.9% of GPs (*N* = 102) but 93.5% of pharmacists (*N* = 187) agreed that there was a demand from patients for vaccination in pharmacies. Nonetheless, in order to avoid multiple vaccinations by different HCPs, 91.0% of both groups (*N* = 456) agreed that it would be necessary to have a tracking system in place to ensure effective coordination of the vaccination programme.

Using the pre-retirement preventive medicine appointment for communicating about pneumococcal vaccination was not the preferred measure for either GPs or pharmacists (overall acceptability score: 6.1 ± 2.5 for both groups combined). Overall, 76.8% of HCPs (*N* = 385) agreed that this was the right age to communicate about pneumococcal disease and 68.5% (*N* = 343) agreed that the appointment would be an appropriate communication channel to reach people aged ≥60 years. However, the need for further communication and explanation about this appointment is highlighted by the fact that only 22.2% (*N* = 111) of HCPs considered themselves familiar with it.

## 4. Discussion

Health authorities in France currently recommend vaccination in patients with certain co-morbidities or who are immunocompromised. Nonetheless, awareness of pneumococcal vaccination is very low, coverage remains unsatisfactory, and there are multiple barriers to optimal implementation [8]. The present study was conducted to evaluate attitudes of HCPs to explicitly integrating age into the pneumococcal vaccination recommendations. In general, HCPs were receptive to such a change and around 90% felt that adding age as a criterion would help increase vaccination coverage. In general, the HCPs who were more open to the idea of extending the vaccination recommendations in this way were those who considered themselves better-informed about pneumococcal disease and its prevention.

The proposal to use the 23-valent vaccine given as a single administration to healthy individuals aged ≥65 years was considered acceptable by HCPs. This is considered to be a conservative step to achieve broad coverage of elderly people and the best protection at the least cost to the healthcare system. The 23-valent vaccine is currently the one with the broadest coverage available today and has demonstrated effectiveness against IPD and CAP in older adults [10]. In this context, recent epidemiological studies have reported an increase in the prevalence of serotypes not covered by the 13-valent vaccine [11,12]. In addition, the use of a single administration of the 23-valent vaccine has been reported to be cost-effective compared to other vaccination strategies in several European countries, [13,14,15,16]. This vaccination strategy has been used successfully for several years in the United Kingdom, as well as in a number of other European countries [6].

France is one of the only OECD countries that does not include age as a specific eligibility factor for vaccination against *S. pneumoniae* [6]. As of June 2021, nineteen countries in Europe recommend vaccination of all individuals aged over 60 or 65 years (depending on the country) [6], and, in the majority of these, the cost of vaccination is fully covered by national health insurance. Vaccination of older adults is also recommended in the United States [17] and Canada [18]. These recommendations have been motivated by the need to extend vaccination coverage in this high-risk segment of the population. As pointed out by the Joint Committee on Vaccination and Immunisation in the United Kingdom in their advice on COVID-19 vaccination [19], “*Age-based programmes are usually easier to implement and therefore achieve higher vaccine uptake. An age-based programme is also likely to increase uptake in those with clinical risk factors as the prevalence of these increases with age*.” In most European countries, a single administration of the 23-valent vaccine is recommended, except for patients who are immunocompromised or who have chronic diseases, for whom sequential administration of the two different vaccines is proposed. For these patients, it is important to combine the high immunogenicity of the conjugate vaccine with the broad serotype coverage of the polysaccharide vaccine. In addition, in the light of the future introduction of new pneumococcal conjugate vaccines with a wider valence coverage, it is timely to reassess the pertinence of current recommendations and the perceptions of HCP towards vaccination strategy.

The study found that HCPs do not consider themselves well-informed about pneumococcal infections or pneumococcal vaccination. For this reason, there is a need to accompany HCPs, particularly pharmacists, with targeted information campaigns to help them be more aware of pneumococcal disease in general and its prevention in particular and to encourage them to be more involved in the vaccination strategy. Nonetheless, most participants were aware that pneumococcal infections were potentially serious and that older adults are at particular risk.

A possibility for improving vaccination coverage would be to optimise the vaccination pathway by involving other classes of HCPs such as pharmacists and nurses. The density of pharmacies in France is high and these are readily accessible. Pharmacists are already in the front line for advising patients about vaccination, notably with respect to the older age group which has a relatively high frequentation of pharmacies. Involving pharmacists has been shown to increase vaccination coverage in a number of diseases, including influenza or pneumococcal infections, in several other countries [20,21,22,23]. For these reasons, public health policies in France are encouraging involvement of nurses and pharmacists in vaccination programmes. In 2018, it was decided to open vaccination provision to pharmacists, except for the vaccination of infants. Pharmacists have been able to offer influenza vaccination since 2019 and SARS-CoV2 vaccination since early 2021, and these are currently the only vaccines authorised for delivery in pharmacies. During the 2019–2020 campaign, pharmacists were responsible for 30% of all influenza vaccinations [24]. Rolling out of the programme to other vaccines was postponed during the SARS-CoV2 epidemic but is expected to resume in the near future. In the case of pneumococcal vaccination, pharmacists are very receptive to the idea of becoming involved, with >90% believing that this would be useful and correspond to patient demand. In contrast, less than half of GPs thought that pharmacists were sufficiently well-trained to do this. A recent survey on the acceptability of vaccination by pharmacists in the French general population found that 77% of respondents were in favour of vaccination at the pharmacist’s for adults and adolescents [25]. This finding highlights the acceptability to the general public of extending vaccination to local community health professionals, with the goal of simplifying the process and reducing the burden on GPs and patients alike.

Finally, HCPs considered that pairing pneumococcal vaccination with influenza vaccination would be a useful strategy for improving coverage in older adults. Influenza vaccination in this age group is now well-established in France and is the object of regular communication programmes targeting both HCPs and the general public. In addition, influenza vaccination coverage is relatively high [26]. For these reasons, it could be beneficial to take advantage of appointments for influenza vaccination in order to promote pneumococcal vaccination, for example by the mailing of a care voucher to eligible individuals.

A potential limitation of the study concerns the representativeness of the HCPs who participated in the study. Although efforts were made to ensure representativeness in terms of age, gender and region, participation was voluntary, so it may be that HCPs who agreed to participate felt particularly concerned by, or were particularly interested in, pneumococcal vaccination and, for this reason, were better informed about the disease and more motivated about vaccination than GPs and pharmacists in France in general. Secondly, the themes addressed in the questionnaire were derived from the qualitative interviews and certain important aspects of vaccination may have been missed.

In conclusion, inclusion of age (≥65 years) as an independent risk factor and criterion in pneumococcal vaccination recommendations would be acceptable to HCPs and would, in their view, facilitate identification of patients to be vaccinated. An age-based recommendation, as proposed in many other countries, would simplify the strategy and probably increase coverage. In addition, vaccination could be offered in conjunction with influenza vaccination and also offered in pharmacies. Such measures could potentially limit loss of vaccination opportunities and improve the unacceptably low current pneumococcal vaccination coverage.

## Figures and Tables

**Table 1:** Characteristics of study participants.

	GPs (*N* = 301)	Pharmacists (*N* = 200)	Total (*N* = 501)
Age (years)			
Median [IQR]	55 [44–61]	17 [39–57]	53 [42–60]
Gender			
Women (n, %)	120 (39.9%)	108 (54.0%)	228 (45.5%)
Place of practice			
Community practice	286 (95.0%)		
Clinic	15 (5.0%)		
Independent community pharmacist		131 (65.5%)	
Assistant pharmacist		52 (26.0%)	
Group pharmacist		16 (8.0%)	
Locum pharmacists		1 (0.5%)	
Region			
Greater Paris region	46 (15.3%)	32 (16.0%)	78 (15.6%)
West	58 (19.3%)	38 (19.0%)	96 (19.2%)
South-West	64 (21.3%)	44 (22.0%)	108 (21.6%)
South-East	67 (22.2%)	44 (22.0%)	111 (22.2%)
North and East	67 (22.2%)	42 (21.0%)	109 (21.8%)

**Table 2:** Acceptability of different measures to accompany extension of the vaccination criteria to include age.

	GPs (*N* = 301)	Pharmacists (*N* = 200)
Use of the pre-retirement preventive medicine appointment “Grow old in good health” in order to build awareness of pneumococcal vaccination		
Overall acceptability score Strategy would be expected to improve vaccination coverage Strategy is relevant Strategy is credible Strategy would be easy to implement	6.1 6.4 ± 2.5 6.2 ± 2.5 6.2 ± 2.5 5.6 ± 2.4	6.1 6.2 ± 2.6 6.3 ± 2.5 5.7 ± 2.6 6.0 ± 2.5
Integration of the pneumococcal vaccination programme into the influenza vaccination programme (with delivery of a vaccination voucher)		
Overall acceptability score Strategy would be expected to improve vaccination coverage Strategy is relevant Strategy is credible Strategy would be easy to implement	8.2 8.3 ± 1.8 8.1 ± 2.0 8.1 ± 1.9 8.2 ± 1.8	8.6 8.9 ± 1.2 * 8.6 ± 1.6 * 8.3 ± 1.6 8.6 ± 1.6 *
Take advantage of the recently-introduced role of pharmacists in influenza vaccination to use them to advise elderly adults about vaccination and to administer the vaccine		
Overall acceptability score Strategy would be expected to improve vaccination coverage Strategy is relevant Strategy is credible Strategy would be easy to implement	5.7 6.0 ± 2.7 5.2 ± 2.7 5.2 ± 2.6 6.4 ± 2.5	8.5 8.7 ± 1.6 * 8.5 ± 1.9 * 8.4 ± 1.8 * 8.3 ± 1.8 *

* Significant (*p* < 0.05) difference between GPs and pharmacists. Data are presented as mean scores ± their standard deviations.

## Data Availability

Requests for access to data, specifying the proposed use, can be made to the corresponding author.

## Supplementary Materials

The following are available online at https://www.mdpi.com/article/10.3390/geriatrics7010004/s1, Table S1: Perceived advantages of including age as a criterion in vaccination recommendations and perceived barriers to implementation.
