# Supplementary material for: The Health Professional’s View on the Inclusion of Age in the Recommendations for Pneumococcal Vaccination: Results of a Cross-Sectional Survey in France
Source: Geriatrics (Basel). 2021 Dec 23;7(1):4. doi: 10.3390/geriatrics7010004 (PMC8788281; doi:10.3390/geriatrics7010004)
Supplement: Supplementary file 1 [file geriatrics-07-00004-s001.zip › geriatrics-1488066-supplementary.pdf]

Table S1. Perceived advantages of including age as a criterion in vaccination recommendations and perceived barriers to implementation

	GPs (N = 301)	Pharmacists (N = 200)	Total (N = 501)
Perceived advantages of the change			
Improve vaccination coverage	73	70	72
Reduce circulation of the pathogen	63	61	62
Provide a public health benefit	61	60	61
Prevent IPD-associated hospitalisations and deaths	60	60	60
Simplify the vaccination schedule	62	56	59
Encourage systematic vaccination	61	46	55
Facilitate recall of the vaccination recommendations	54	51	52
Provide an individual benefit	46	49	47
Perceived barriers to implementation			
Vaccine hesitancy in a segment of the French population	59	54	57
Patients do not consent	58	42	51
Cost of extending reimbursement to a new segment of the population	32	39	35
Availability/stocks of vaccine	27	35	30
An elderly population already targeted for other types of vaccination	25	24	25
Poor acceptability of the vaccine in elderly people	23	16	20
Elderly people in good health may not need to be vaccinated	19	21	20
Lack of time to discuss vaccination during consultations	11	11	11

Data are presented as the proportion of participants selecting the item from a pre-specified list. IPD: invasive pneumococcal disease.
